# Individual EEG measures of attention, memory, and motivation predict population level TV viewership and Twitter engagement

**DOI:** 10.1371/journal.pone.0214507

**Published:** 2019-03-28

**Authors:** Avgusta Y. Shestyuk, Karthik Kasinathan, Viswajith Karapoondinott, Robert T. Knight, Ram Gurumoorthy

**Affiliations:** 1 R&D department, Nielsen Consumer Neuroscience, Berkeley, California, United States of America; 2 Helen Wills Neuroscience Institute, University of California, Berkeley, California, United States of America; 3 Department of Psychology, University of California, Berkeley, California, United States of America; La Sapienza University of Rome, ITALY

## Abstract

Television (TV) programming attracts ever-growing audiences and dominates the cultural zeitgeist. Viewership and social media engagement have become standard indices of programming success. However, accurately predicting individual episode success or future show performance using traditional metrics remains a challenge. Here we examine whether TV viewership and Twitter activity can be predicted using electroencephalography (EEG) measures, which are less affected by reporting biases and which are commonly associated with different cognitive processes. 331 participants watched an hour-long episode from one of nine prime-time shows (~36 participants per episode). Three frequency-based measures were extracted: fronto-central alpha/beta asymmetry (indexing approach motivation), fronto-central alpha/theta power (indexing attention), and fronto-central theta/gamma power (indexing memory processing). All three EEG measures and the composite EEG score significantly correlated across episode segments with the two behavioral measures of TV viewership and Twitter volume. EEG measures explained more variance than either of the behavioral metrics and mediated the relationship between the two. Attentional focus was integral for both audience retention and Twitter activity, while emotional motivation was specifically linked with social engagement and program segments with high TV viewership. These findings highlight the viability of using EEG measures to predict success of TV programming and identify cognitive processes that contribute to audience engagement with television shows.

## Introduction

Today, we experience what many have dubbed a “TV Renaissance,” with an increasing number of television (TV) shows of varying quality produced by traditional networks, including broadcast and cable, and by online streaming companies, such as Netflix, Amazon, and Hulu [1]. In this sea of the ever-expanding TV content, it becomes exceedingly important to know what makes TV shows good. Television success has been traditionally measured in terms of viewership (i.e., how many people watch each show episode at any given moment or overall) [2–4]. However, with the advent of social media and the rising popularity and reach of social media websites such as Facebook, Twitter, and Instagram, viewers’ social online engagement with TV shows (e.g., sharing viewership preferences, reacting to show content, etc.) has become another important measure of show success [2, 5–8]. The “eyes-on-the-screen” viewership metric increasingly gives way to “likes-on-the-page” indicators, as TV show retention and cancellation may depend on factors that go beyond traditional viewership ratings, including show’s presence on social media in the form of tweets, likes, and shares [7–9]. Several recent studies have examined patterns of social media use (in particular, Twitter) surrounding TV shows such as Walking Dead [10], Downton Abbey [9], or reality and news TV [11–13]. These studies suggest that social media engagement with a particular TV show is often driven by two major categories of factors: internal (e.g., self-presentation or sense of social connectedness) and content-driven (e.g., emotional responses, preferences, or opinions about show content) [9–13]. Similar research also indicates that the volume and content of show-related tweets correlate with show content and can be effective in assessing TV show success [2].

As audiences migrate their discussions about favorite shows away from watercoolers to social media websites and apps, show-runners (e.g., media executives, producers, show creators, directors, and writers) not only monitor social media presence of their shows but also actively promote show-specific social media communities and catalyze show-centric online chatter by using Twitter hashtags, maintaining active Facebook/Instagram/Twitter profiles, and encouraging show stars to engage with social media [2, 7–8, 13]. The reason for such an extensive symbiosis between TV content and social media is the reciprocal relationship between show-specific social engagement and TV viewership. Social ‘buzz’ around the show, serving as an effective advertisement, can drive the overall viewership of future episodes as well as active, real-time audience participation [8, 14–16]. Thus, the new strategy of show-runners is to increase viewership through social media engagement, which could foster greater popularity and, ultimately, translate into higher revenue from both distribution and advertisement [7–8].

Although Twitter and other social media activity has become an important indicator of TV show performance as it airs, accurately predicting future success of a new TV show or upcoming show episodes is still quite a daunting task–complex predictive models are often employed to gauge potential success of a TV show, including such variables as genre, target demographic, market saturation/paucity, strength of existing competition, seasonality, and distribution/broadcast options [17]. But the main driver of television success remains the strength of the creative content–audiences want to watch great shows that are emotionally evocative, memorable, and inspiring [2, 9–12, 18–20]. Capturing this “lightning in the bottle” is challenging since it is often difficult to predict which creative idea will resonate well with viewers and which will fall flat [21–23]. Thus, it becomes increasingly important for show creators, producers, and media executives to optimize the creative content in order to maximize viewership potential and social reach.

Commonly employed assessments of TV show quality involve techniques that are based on subjective opinions of potential viewers (e.g., focus groups, self-report questionnaires, and rating dials) [2, 9–10, 22–23]. However, such techniques suffer from several significant shortcomings [24–27]. First, reporting biases may skew focus group and questionnaire results since viewers often tailor their expressed opinion based on the context of the interaction (e.g., how the questions are phrased, who is leading the focus group, or how the other members of the group respond). Second, viewers often have difficulty reporting which elements of the show they find particularly engaging and which parts they dislike or are indifferent about. The overall impression can overshadow or accentuate viewers’ perceptions of specific parts of the show. Finally, when rating dials are used to evaluate moment-to-moment fluctuation in viewer perceptions, results can be confounded by the dual-tasking nature of the technique and are limited in scope as only one dimension can be examined at a time [28].

In recent years, the entertainment and advertising industries have seen an emergence of alternative techniques to evaluate creative content, including functional magnetic resonance imaging (fMRI) and electrophysiological recordings, which rely on direct measurements of viewers’ brain activity as they watch movie clips, movie trailers, TV programming, or commercial advertisement [29–46]. These neuroscience-based techniques are less susceptible to self-report biases and, thus, represent a more accurate and direct assessment of viewers’ responses to creative content [47–51]. Some neuroscience measures, such as electroencephalography (EEG) and electrocorticography (ECoG), are also characterized by exceptional temporal resolution, which allows for a more granular, moment-to-moment measurement of viewers’ responses [32, 44, 50–52].

There have been several fMRI, EEG, and ECoG studies evaluating brain activity in response to creative content, including short movie clips [40] or excerpts from popular movies [34, 38–39, 46]. These studies have been able to identify distinct cortical clusters, primarily in the sensory (audio and visual) cortical areas, that reliably respond to complex naturalistic stimuli and preferentially tune to specific content features of the videos (e.g., faces or action/movement). Although these results demonstrate that brain activity can be successfully used to study naturalistic video stimuli, none of the above studies have compared brain activity patterns against a specific objective or subjective preference criterion (e.g., box office performance, participant preference, etc.). Such criterion validation was the aim of a recent study that investigated relative efficacy of fMRI and EEG measures in predicting performance of TV advertisement, quantified as a change in market share of advertised products [45]. Results reveal that increased fMRI activity in the amygdala, dorsolateral prefrontal cortex, ventral striatum, and ventromedial prefrontal cortex (brain areas associated with emotional and cognitive processing) were most predictive of advertisement in-market performance when added to the traditional self-report measures [45]. However, when all independent measures were assessed in isolation, a combined EEG measure was superior in predicting in-market ad performance relative to fMRI, accounting for 34% of explained variance. In this study, the combined EEG measure was a composite of frontal left-to-right asymmetry in the alpha frequency band (~8–12 Hz; often linked across studies with positive emotional states and approach motivation [53–62]) and a decrease in occipito-parietal alpha power (commonly associated with sensory attentional processing [63–72]). These findings highlight efficacy of EEG-based techniques in assessing viewers’ preference for creative content of complex naturalistic stimuli.

The observed predictive utility of EEG metrics has been confirmed by several other studies that have explored the link between EEG-derived measures of brain activity and empirically established effectiveness of media and TV advertisements [29–33, 36, 42–44, 55, 66, 73–81]. For example, researchers have been able to predict preferences for movie trailers as well as eventual box office success of the corresponding movies using either a single composite EEG score (similarity index) [29] or power within individual frequency bands, such as beta (~13–25 Hz) and gamma (~30–55 Hz) [30–31]. Similarly, several studies have observed correlations between EEG measures and self-reported TV show preference [43] or online view counts for Super Bowl ads [32]. While these studies examine a plethora of different EEG signatures, a common thread has emerged, suggesting that advertisement effectiveness and processing of naturalistic video stimuli depend on cognitive processes of attention, memory, and emotion, the relative weight of which varies with the outcome variable, such as ad memorability or subjective preference [33, 42–45, 80–81].

Specifically, a decrease in occipito-parietal alpha power has been typically associated across a variety of consumer neuroscience studies with increased alertness and sensory attention [63–64, 67]. Decreases in posterior alpha power have also been found to track visual scene changes [66, 72–74] and subjective arousal [73]. In contrast, fronto-centrally distributed decreases in alpha power have been linked to perceived interest in ad content and subsequent ad memorability, suggesting that fronto-central alpha power is more sensitive to cognitive aspects of attentional focus than parietal alpha [67, 74]. Another EEG metric used to evaluate creative content is left-to-right frontal alpha asymmetry, with greater decrease in alpha power over the left relative to right hemisphere correlating with approach motivation, often expressed as positive affect [33, 41, 53–61, 76]. This greater left-to-right frontal alpha lateralization has been observed in response to positively judged TV content [55], positively perceived movie clips [59–61], and has been linked with activation of the dopaminergic reward system [79–80] as well as self-reported levels of behavior activation [81–82]. Thus, it has been proposed that the alpha asymmetry serves as a biomarker of the behavioral activation system (BAS), as proposed by Gray [81–84]. Specifically, the alpha asymmetry is thought to extend beyond positive affect or valence and reflect a broad trait or state behavioral and motivational approach (as opposed to inhibition) [59, 62, 85–87]. Finally, Vecchiato and colleagues have examined EEG correlates of ad memorability across a number of studies, demonstrating that subsequently recalled TV ads are characterized by power increases in the fronto-central theta (~4–8 Hz) and gamma frequency bands during encoding (either as a single composite EEG score–impression index–or as individual frequency band measures) [36, 76–77]. These results are in line with other reports of greater theta and gamma power over the fronto-central regions during successful memory encoding and recollection of various types of stimuli [88–94], confirming the viability of these EEG measures in examining memory-related processes during viewing of naturalistic stimuli, such as TV shows [76–77].

In combination, these results suggest that emotional motivation, memory, and attention are all important factors contributing to favorable perception of complex naturalistic stimuli, such as video advertisements or movie excerpts and trailers [33, 76–77]. These factors can be used to predict real-world success and relevance of naturalistic stimuli [29–32] and are likely to represent success predictors of TV programming–shows have to be memorable, attentionally engrossing, and emotionally engaging to prompt viewers to stay with the program or return for more episodes. These cognitive processes, especially emotional motivation, are also likely to compel viewers to share personal impressions of the show via social media, given preliminary research demonstrating that the desire to share information is associated with activity in brain areas linked with emotional processing, including the theory of mind and social perception (i.e., the medial prefrontal cortex and temporo-parietal junction [95–96]).

Although there have been a number of studies, as described above, that have used EEG metrics to examine efficacy of TV commercials or assess brain activity during watching movies and video clips, few studies have applied EEG methodology to examine viewership or social media engagement of TV shows. In a recent study, Fischer and colleagues correlated frontal EEG asymmetry with whether participants were more or less likely to “share” a short video in a laboratory context, approximating sharing behavior on YouTube or Facebook platforms [97]. While this study had a low number of participants and a high number of tested stimuli, it revealed that shared videos also elicited greater decreases in alpha power over occipital and fronto-central sites, potentially indicating greater visual and cognitive attention to stimuli that are deemed worthy to be shared. A different, “second screen”, study has examined cognitive engagement (as indexed by the Steady State Topography—SST) while participants watched a live reality TV show and were free to use social media, including Twitter [98]. The authors reported increased SST-measured engagement during second screen activity periods (as participants were tweeting) as well as an increase in engagement during subsequent TV show segments. Although the increased engagement during live tweeting may be explained by the dual-task nature of the activity as well as greater cognitive load during active tweeting relative to passive viewing, these results indicate that social media participation during a live TV show may increase interest and promote viewership for the entire episode. Finally, the sole study that has examined the relationship between EEG-measured brain activity and moment-by-moment fluctuations in TV viewership and Twitter activity in participants watching a single TV episode (the premier episode of “The Walking Dead” TV series) found that global synchronization of EEG activity across participants watching the TV program was correlated with both TV viewership and Twitter volume measured for the audience as a whole [99]. This single EEG measure of cross-subject synchronization is conceptually similar to increases in BOLD signal and is in line with previous fMRI and ECoG studies demonstrating similarities across participants in sensory brain areas (i.e., temporal and occipital) when processing complex naturalistic stimuli such as movies or video clips. Thus, EEG synchronization observed in this study was likely driven by attention to common auditory and visual features of the stimulus [38–40, 46].

Taken together, these studies suggest that TV viewership and social media engagement with a particular creative content can be estimated using EEG-derived measures. However, given the lack of generalizability among existing research (as only a single episode of a TV show [98–99] or YouTube videos with low view numbers [97] have been explored), several key questions remain. First, none of the studies have examined whether TV viewership and Twitter engagement depend on specific cognitive processes elicited by TV content. In particular, as discussed above, multiple reports highlight the role of emotional motivation, attention, and memory in viewer preference for TV content [33, 42–45, 80–81]. Thus, it is likely that these factors also contribute to episode-specific indicators of TV show success such as viewership and Twitter volume. Second, both single composite EEG metrics and individual EEG measures based on specific frequency bands have been employed in previous studies without a clear analysis of whether these metrics are comparable in their predictive ability. Finally, it remains under debate whether EEG measures can add additional predictive power beyond traditional measures and explain more variance in TV viewership and Twitter activity than other behavioral variables [45].

The current study aimed to address these questions and to determine whether population-level success (as expressed in total TV viewership and Twitter volume during live program broadcasts), of a broad range of TV shows (9 episodes from 8 prime-time TV shows) could be predicted using a single composite EEG score or EEG-derived metrics of attention (as indexed by fronto-central alpha decreases and concomitant theta increases [33, 63–64]), memory (as indexed by the fronto-central power increases in the theta and gamma frequency bands [36,76–77]), and emotional motivation (as indexed by the left-to-right asymmetry in the alpha and beta frequency bands [33, 53]). The average magnitude of these EEG measures, recorded from an independent sample of participants within each contiguous show segment (recorded in-between commercial breaks; n = 49), was correlated with the average TV viewership (the overall number of people watching the program, sans commercials, when it first aired) and the overall number of show-related tweets posted by Twitter users during the original episode broadcast. Thus, we used EEG measures derived from an independent set of participants who did not watch the original episode broadcast and who experienced the episode narrative for the first time during the EEG session to predict the two population-level metrics of TV viewership and Twitter activity. We hypothesized that all three cognitive processes would be critical predictors of viewership and tweet numbers, on par with a single composite EEG score. In addition, given that Twitter activity has been linked with social motivation and is often seen as driven by emotional content of TV programming [9–13], we hypothesized that Twitter engagement may be more correlated with the fronto-central EEG asymmetry than TV viewership.

## Methods

### Participants

#### EEG study

331 individuals were recruited from the San Francisco, Chicago, and Atlanta metropolitan areas. All prospective participants were screened for neurological, psychiatric, and other medical disorders and conditions that are known to affect cognitive functioning or to alter frequency or amplitude of EEG signals. Individuals routinely taking any psychoactive medications (prescription, over the counter, or recreational) were excluded. Prospective participants were also asked to complete a handedness questionnaire and tests of vision acuity and colorblindness. To minimize across-subject variability, only right-handed individuals with normal or corrected to normal eyesight qualified for the study.

To control for familiarity with the content of the tested TV episodes and to ensure episode comprehension, only those individuals who regularly watched a given TV show (but did not see the tested episode) were included in the study. A representative sample was created by including individuals who used Twitter regularly, sometimes, or never. On average, 77.1% (*SD* = 9.5%) of participants had Twitter accounts, regularly posting tweets (58.4%, *SD* = 18.0%) or reading tweets (66.6%, *SD* = 13.4%); 25% (*SD* = 10.9%) of participants never used Twitter, although some of them had active accounts.

Based on their TV show habits and preferences, qualified participants were assigned to one of nine cells, each corresponding to a given TV episode. Data for each episode were collected from 36–38 participants, with equal number of males and females. Participants were 21–54 years old, with equal distributions across the low (21–34 years old) and high (35–54 years old) age ranges in each cell. Race and ethnicity composition of the sample were kept consistent with the latest national census data. All participants were asked to read and sign an informed consent document prior to testing. After the study completion, participants were debriefed and remunerated. All procedures were carried out in accordance with protocols approved by an external Institutional Review Board–Ethical and Independent Review Services (Corte Madera, CA).

#### Twitter volume

Minute-by-minute Twitter volume data (i.e., total number of tweets) for each TV episode were obtained from the Twitter TV Ratings, provided by Nielsen Social (part of the Nielsen Company, New York, NY; www.nielsensocial.com) through a direct agreement with Twitter (San Francisco, CA). Tweet data were obtained and analyzed in accordance with Twitter policies on data privacy and sharing; only anonymized and aggregated Twitter volume values were used. Twitter volume data represented all tweets posted by episode viewers in real time during the initial broadcast of the episode.

#### TV viewership

Minute-by-minute TV viewership estimations were obtained as volume totals from the Nielsen TV Ratings database (www.nielsen.com). Only anonymized and aggregated viewership totals were used in this study. TV viewership data represent the estimated number of individuals who watched the episode in real time as it aired.

### Stimuli

Selected TV shows (Table 1) had comparable characteristics and were representative of the current TV landscape. Only hour-long prime-time serial shows that were airing new episodes at the time of the study were used. Eight shows (nine episodes) were originally chosen to provide a representative range (low to high) of Twitter engagement, as measured by the Nielsen Twitter TV Ratings, and TV viewership, as measured by the Nielsen TV Ratings (see Table 1). Show selection was performed using Twitter volume and TV viewership data for all episodes of each show aired prior to the selection process and was aimed to ensure relative independence between measures of Twitter engagement and TV viewership–although the Big Brother episodes drew the most tweets and largest audiences. Since recruitment age for the EEG portion of the study was limited to 21–54 y/o, shows for which over 50% of viewers or Twitter users were outside of that age range were excluded to ensure demographic commonality among each show’s TV audience, Twitter users, and EEG study participants.

**Table 1:** TV episode information and pre-processing details.

Show	Network	Network type	Show type	Average Twitter volume^a^	Average TV viewers^a^	Twitter lag^b^	Omit start^c^	Omit end^d^
**Big Brother Ep. 1**	CBS	Broadc.	Reality	979.0	3,345,932	2	3	2
**Big Brother Ep. 2**	CBS	Broadc.	Reality	502.3	4,109,862	0	2	1
**Gang Related**	Fox	Broadc.	Drama	66.2	1,288,583	2	0	0
**MasterChef**	Fox	Broadc.	Reality	101.7	2,825,495	1	3	2
**Naked and Afraid**	Disc.	Cable	Reality	58.1	1,205,048	0	3	2
**NY Med**	ABC	Broadc.	DS	41.4	4,025,144	1	0	0
**Reckless**	CBS	Broadc.	Drama	30.2	2,897,098	2	2	2
**Suits**	USA	Cable	Drama	120.4	1,438,171	1	2	2
**Taxi Brooklyn**	NBC	Broadc.	Drama	22.0	3,324,714	1	2	3

Broadc.–broadcast, Ep.–episode, Disc.–Discovery, DS–documentary series.

^a^ For each episode, numbers of tweets and TV viewers were calculated for each minute of the show and then averaged across the show duration.

^b^ *Twitter lag* (in minutes) was used to align minute-by-minute Twitter volume values with EEG and TV viewership values, correcting for the lag between what people see on the screen and when they type and post tweets online. For example, a lag of 2 minutes means that tweets from minute (*t+2*) are aligned with EEG and viewership values for minute *t*.

^c^ *Omit start* (in minutes) reflects the number of minutes at the beginning of each show that were omitted from analyses due to outlier Twitter or viewership values (audiences tuning in late or tweeting about the show in general rather than about the content of the specific episode).

^d^ *Omit end* (in minutes) reflects the number of minutes at the end of each show that were omitted from analyses due to outlier Twitter or Viewership values (audiences for the next show tuning in early or tweeting about the upcoming episode rather than about the content of the current episode).

Out of the eight selected shows, six aired on network TV, and two shows aired on cable TV. Three shows were reality or competition programs (e.g., *MasterChef*), four shows were dramas (e.g., *Suits*), and one show was a documentary serial show (*NY Med*). For one of the shows, *Big Brother 16*, two episodes were used, as the episode originally selected for testing was aired 2 hours later on the East Coast due to a delay in sport programming preceding the episode. The replacement episode of *Big Brother 16* was also delayed (by 51 mins) on the East Coast for a similar reason. Since West Coast air times were not affected for either episode, and given that Twitter volume and TV ratings for these two episodes did not appear to be significantly different from previous episodes of the show, we decided to include both episodes in the final analyses, bringing the total number of tested TV episodes to nine. Episodes were selected to allow EEG data collection to be completed within 3–4 days after the initial broadcast to minimize potential exposure to the tested episode prior to the EEG session. All tested episodes came from the second half of their respective seasons, which made it easier to recruit participants who were familiar with the show and watched it on a regular basis.

For the EEG portion of the study, each episode was recorded in high definition (1366x768) when it originally aired and was edited off-line to remove commercial breaks. Commercial breaks were omitted to remove potential cross-influences of advertisement content on the overall cognitive and emotional processing of the subsequent show segments [100–102]. In addition, our goal was to maximize comparability between EEG measurements and content-specific Twitter activity and TV viewership. Thus, we used uninterrupted episodes for the EEG portion of the study to provide a close match with Twitter and audience measurements.

### Procedures

For each episode, participants for the EEG study were recruited prior to the scheduled on-air date and were asked not to watch that particular episode or read/watch media coverage related to it before the experimental session, which was scheduled 2–4 days after the original air date. Upon arrival to the lab, each participant was asked to read and sign the informed consent form, after which they were led to a separate prep area, and a trained technician applied EEG and auxiliary electrodes. During the experimental session, each participant was seated in a private soundproof room with comfortable lighting. Episodes were presented without commercial interruptions on a 42” TV screen positioned 5 feet away from the participant’s chair. A post-session questionnaire was used to verify that participants had not previously watched the episode or heard about it from family, friends, or traditional and social media. Each experimental session lasted about 1 hr 15 mins. Given each lab’s capacity to collect 3–5 participants simultaneously (in separate sound-proof booths), data from all participants for each TV episode were collected within 2–3 days (up to 4 days post original show broadcast). This short fielding duration minimized potential influences of time elapsed since the initial episode broadcast and ensured that participants within each group had similar familiarity with the previous episode and did not have a chance to see the next one. Twitter volume and TV viewership data were obtained from the Nielsen Company within a week after the original on-air date for each episode.

### EEG recordings and apparatus

Continuous EEG data were collected with a 512 Hz sampling rate using a Biosemi (Amsterdam, Netherlands) ActiveTwo DC-coupled amplifier and a biopotential measurement system with 32 active scalp electrodes [103–104] referenced to the linked CMS-DRL electrodes. Data were digitized at 24 bit per channel, with 31nV LSB. Acquisition software filters (5^th^ order sinc response) were set at 0.1 Hz high-pass and 250 Hz low-pass. Additional surface electrodes above and on the side of the left eye were used to measure vertical and horizontal eye movements. Electrodes with bad connections were substituted with Biosemi external sensors prior to data collection, resulting in loss-free data. To remove common system noise and artifact, EEG signals were re-referenced offline to the external electrode positioned at the tip of the nose (to minimize muscle and eye movement contaminations). EEG signal acquisition and data analyses were performed in compliance with the established practices and guidelines for EEG research [103].

### Data analyses

#### EEG

All analyses were completed using the Matlab software (Mathworks, Natick, MA). During EEG preprocessing, muscle noise and eye movement artifacts were corrected using independent and canonical component analysis algorithms adapted from EEGLab functions [105], in which components associated with eye movements and muscle noise were isolated and removed from the raw data [106–107]. Success of artifact correction was visually confirmed by trained signal processing engineers. Data segments with residual contaminants were excluded from subsequent analyses. Participant data containing excessive residual contamination were excluded. The exclusion rate was about 35% due to long-format programming (continuous stimulus over 40 min in length) and short turn-around time for recruitment and data collection to avoid pre-session exposure to the episode. Potential data loss was expected, and initial recruitment goals were set with that in mind. Thus, final analyses for each episode were performed on clean data from 23–24 participants.

EEG data from each channel were subjected to the spectral decomposition, in which power was extracted within pre-defined frequency ranges for each 1000 ms non-overlapping segment using the short-time Fourier transform (stft) [108]. In particular, spectral power (stft-extracted analytic amplitude squared) was calculated in the following frequency bands: theta (4–8 Hz), alpha (8–12 Hz), beta (13–30 Hz), and gamma (31–55 Hz). Second-by-second power values within each frequency band were baseline corrected to the 1,000 ms window, corresponding to a black screen immediately preceding TV episode onset, and averaged across 15 frontal and fronto-central sites (left: Fp1, AF3, F7, F3, FC5, FC3; midline: Fpz, Fz, FCz; right: Fp2, AF4, F4, F8, FC4, FC6), creating a composite power estimation, which was then normalized by converting power values to z-scores across time within each participant [33, 36, 76, 103, 109]. This procedure produces global region-of-interest power estimations within each frequency band that are more robust than deriving and examining power at individual sites [33, 36, 76]. For the fronto-central asymmetry measure, detailed below, power values were averaged across right and left sites separately [54].

Obtained z-scored power values were combined to create three EEG scores. First, a relative power ratio of left-to-right sites was calculated for the alpha (inverse) and combined with relative power ratio in the beta frequency bands (denoting motivational approach) [33, 53–62, 76, 84–87]. Second, power values in the theta and gamma frequency bands (denoting memory processing) were calculated across all preselected sites [36, 76–77, 88–94]. Finally, a decrease in alpha power combined with a concomitant increase in theta power (denoting content-specific attention focus) was calculated across all preselected sites [63–69, 72–74]. For each of the three frequency combinations, second-by-second power values were averaged across participants and scaled using a sigmoid function unique for each frequency combination. The sigmoid transformation was used to minimize potential influences of extreme values (both high and low) and to convert raw power amplitude to values normally distributed along a continuous 10-point scale, which would make results from different frequency bands easier to compare (as the absolute raw power amplitude varies as a function of the frequency of the oscillation) [110]. These second-by-second scores were then averaged for each minute of the program to match temporal resolution of Twitter volume and TV viewership. To provide a more direct comparison between our analyses and other consumer neuroscience research using a single group-aggregate metric (e.g., impression index, neural similarity or neural reliability measures [29, 36, 99]), we computed an additional composite score (an average of the minute-by-minute scores for the three frequency combinations), calculated across time within each participant and then aggregated across participants. Given that the composite EEG score was in equal part derived from the three frequency combinations known to index motivation, memory, and attention, this metric could be seen as a reflection of the overall cognitive and motivational engagement with TV programs at the group level.

Since Twitter, TV viewership, and EEG data fluctuate at different scales, we hypothesized that the rate of change (i.e., minute-to-minute differences in values. V_t+1_-V_t_) would be a more appropriate metric than the raw amplitude values alone. Thus, EEG scores were converted to the difference scores, which were then averaged within each continuous program segment originally shown between commercial breaks. Each episode yielded 5 to 6 segments, depending on the number of commercial breaks (EEG was not available for the last segment of the *Suits* episode due to an error in video editing). In total, 49 program segments were analyzed. The goal of segmentation was to minimize random minute-to-minute fluctuations in brain activity [111], Twitter volume, and TV viewership. Those minutes when an episode transitioned to commercials were allocated as “program” if the duration of the episode segment was more than 30 seconds, otherwise such minutes were counted as part of the commercial break.

#### Twitter volume

The Twitter volume measure included all tweets matching pre-defined show- and episode-specific keywords derived by the Nielsen Social group to estimate Twitter TV ratings [10, 13]. Per standard Nielsen Social methodology widely used by TV networks, both tweets and re-tweets were included. Tweets from all time zones were aggregated after the appropriate time adjustments. Promotional tweets from the network or those related to advertised products within the show commercial breaks were excluded. For the duration of the episode, the Twitter volume was quantified on a minute-by-minute basis as a number of all tweets within each minute of the show. Within each episode, Twitter volume values were normalized by converting them to z-scores in order to remove between-episode variability in Twitter volumes (as seen in Table 1) and to match EEG data analysis procedures (see above).

We observed that Twitter volume data considerably fluctuated at the beginning and at the end of programming, often resulting in extreme outlier values that did not reflect episode-specific content (e.g., early tweets reflecting the overall excitement about the upcoming episode or end-of-show tweets reacting to the whole show or anticipating next-week’s episode). The prevalence of content-independent anticipation and wrap-up tweets at the start and end of TV shows has been previously reported in several studies of TV-related Twitter use dynamics [9–10]. Given that these extreme values are not related to the immediate episode content and, thus, skew Twitter volume estimations for the beginning and the end epochs of each show, we omitted these values from analyses, optimizing omission periods (1–3 minutes at the beginning and the end) for each program (Table 1). The corresponding minutes were also omitted from the EEG and TV viewership data before segmentation.

Next, we considered the fact that there is an inherent lag between events of the program and when tweets are posted online. In particular, while the lag between events on the screen and EEG activity in response to these events is on the order of milliseconds, the lag between on-screen events and Twitter activity is likely to be on the order of seconds-to-minutes and could vary from show to show depending on the structure and content of each episode [2, 9–10]. For example, not counting the delay between events in the show and the decision to tweet, it could take about 30 seconds to write and post an original tweet, while retweeting might take only a few seconds. Given the variation in original twitter length (mean = 66.03, *SD* = 5.66; ranging from 59.63 for *Big Brother 1* to 75.97 for *Reckless*) and the ratio or original tweets to retweets (mean = 2.60, *SD* = 1.40; ranging from 0.87 for *Taxi Brooklyn* to 4.89 for *Naked and Afraid*), we assessed lags of 0 to 2 minutes and selected episode-specific lags that were the most reflective of temporal dynamics of tweeting for that episode (Table 1).

**Fig 1:**
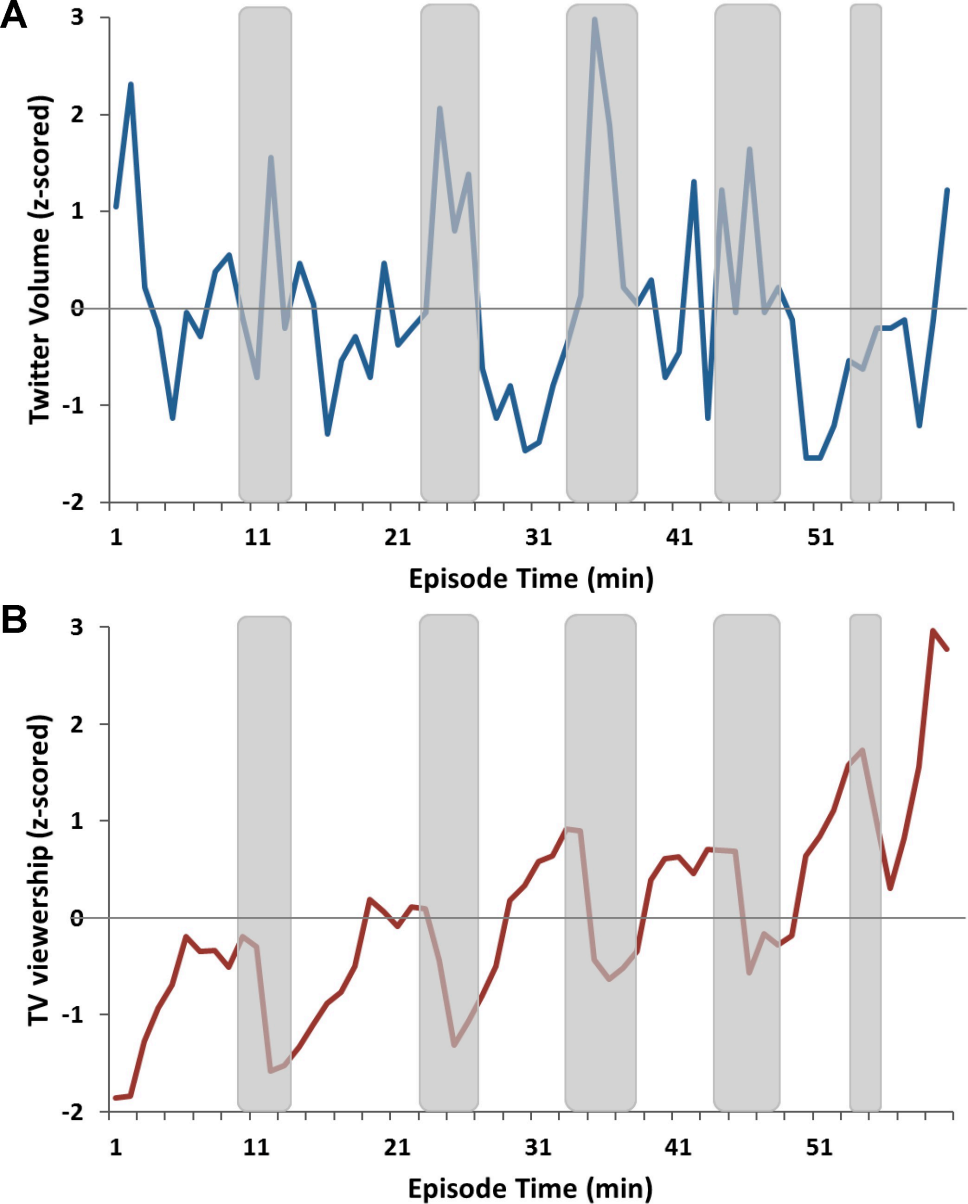
Minute-by-minute changes in Twitter volume and TV viewership across episode duration. (A) Minute-by-minute changes in Twitter volume for a sample episode (*NY Med*). Shaded areas denote commercial breaks. Twitter activity increased during commercial breaks relative to show segments. (B) Minute-by-minute changes in viewership for a sample episode (*NY Med*). Shaded areas denote commercial breaks. TV viewership decreased during commercial breaks, with subsequent rebound during the next show segment. These patterns of Twitter and TV viewership fluctuations across the show duration were typical for most tested TV episodes; *p* = 0.03 for Twitter volume; *p* < 10^−5^ for TV viewership.

Finally, Twitter volume typically increases during commercial breaks [10, 12], which represents the remnant impact from the preceding program segments as people tweet about the content of the previous show segment during breaks in the narrative (Fig 1A). With the exception of the first show segment of *Taxi Brooklyn*, which was dominated by promotional network tweets, all other show segments (n = 48) across all episodes saw a decrease in content-related Twitter activity relative to commercial breaks (n = 44); *t* (90) = 2.21, *p* = 0.03. Thus, to account for the increase in content-related Twitter volume during commercial breaks, we added tweets from the first two minutes of a commercial break to the segment average. After correcting for the temporal lag, accommodating delayed tweet activity during commercial breaks, and removing beginning and end extremes, the minute-to-minute changes in z-scored Twitter volume were averaged within each continuous segment between commercial breaks to match EEG data segmentation.

#### TV viewership

The TV viewership was quantified using Nielsen TV Ratings data, which estimates the number of viewers per minute for the entire household during the show airtime calculated across time zones (Table 1). To match Twitter data pre-processing, TV viewership data were also subjected to z-score normalization within each episode to control for inter-episode variability in TV viewership. One-to-two minutes (optimized by program) were excluded from the beginning and the end of each show to account for late arrivals and hold-over viewers from the previous program at the beginning of each show as well as early drop-offs and early tune-in audiences for the next show at the end of the program (Table 1). Further, it has been widely reported that commercial breaks experience audience drop-off relative to the programming content, which can, in turn, affect viewership volume for the subsequent program segment [112–114]. This drop-off was also apparent in our data, with program segments exhibiting higher TV viewership than commercial pods (*t* (91) = 4.83, *p* < 10^−5^, n_ShowSeg_ = 49, n_AdBreaks_ = 44; Fig 1B). The magnitude of the commercial pod drop-off depends on multiple factors, including advertisement content and quality, commercial pod length and serial position within the program, program genre, program day and time [114]. Thus, to accommodate the TV viewership drop-off during commercial pods and the subsequent reduction in TV viewership for the beginning of the following program segment, the difference in TV viewership from the end to the start of the commercial break was subtracted from subsequent segment’s values to remove this trend. Finally, similarly to the EEG and Twitter volume data, the minute-to-minute changes in TV viewership were calculated and averaged within each program segment, including the first minute of the commercial break to compensate for the lag in viewership disengagement (and channel switching) during the transition from the program to the commercial break.

## Results

### Episode-level analyses

Confirmatory top-level analyses were performed across episodes to quantify differences in Twitter activity, TV viewership, and EEG scores. As shows were specifically selected to have a representative spread on both dependent variables (Twitter volume and TV viewership; Table 1), we expected to find significant differences across episodes. We performed two one-way ANOVAs, across all minute-by-minute values (square root normalized), with the main factor representing the 9 episodes. We observed significant differences across episodes for Twitter volume; *F*(8 , 537) = 633.70, *p* < 10^−5^. Post-hoc pair-wise comparisons revealed the following pattern of Twitter activity across recorded episodes: Big Brother 1 > Big Brother 2 > MasterChef = Suits > Gang Related > = Naked and Afraid > = NYMed > = Reckless > = Taxi Brooklyn (all significant *p*-values < 10^−3^, Bonferroni corrected for multiple comparisons; > = indicates that the Twitter volume for the preceding episode was not significantly different from the following episode but was significantly greater than Twitter volume for all subsequent episodes on the list). There were also significant differences in TV viewership among episodes; *F*(8 , 537) = 1538.88, *p* < 10^−5^. Post-hoc pair-wise comparisons revealed the following pattern of TV viewership across episodes: Big Brother 2 = NY Med > Big Brother 1 = Taxi Brooklyn > MasterChef = Reckless > Suits > Gang Related = Naked and Afraid (all significant *p*-values < 10^−3^, Bonferroni corrected for multiple comparisons).

Similar ANOVA analyses were performed on the minute-by-minute EEG scores (power in the fronto-central alpha/theta and theta/gamma frequency bands as well as the alpha/beta asymmetry). Significant cross-episode differences were detected for the alpha/beta asymmetry and the theta/gamma frequency power. Specifically, for the alpha/beta asymmetry, the *NY Med* and *Taxi Brooklyn* episodes had the lowest EEG scores; *F*(8 , 368) = 11.79, *p* < 10^−3^ (these episodes were also among the lowest for Twitter volume). For the theta/gamma power, the *MasterChef* episode had the lowest EEG scores; *F*(8 , 368) = 8.12, *p* < 10^−3^. Since we expected to see top-level differences across episodes on the dependent and independent variables in light of our selection criteria (to have a representative sample of TV episodes) and since the total number of episodes was low and did not allow for comprehensive analyses at this level, all further analyses were performed on normalized data (removing cross-episode differences) averaged within contiguous show segments (removing and/or pre-allocating data from commercial breaks; see Methods).

### Segment-level analyses

**Fig 2:**
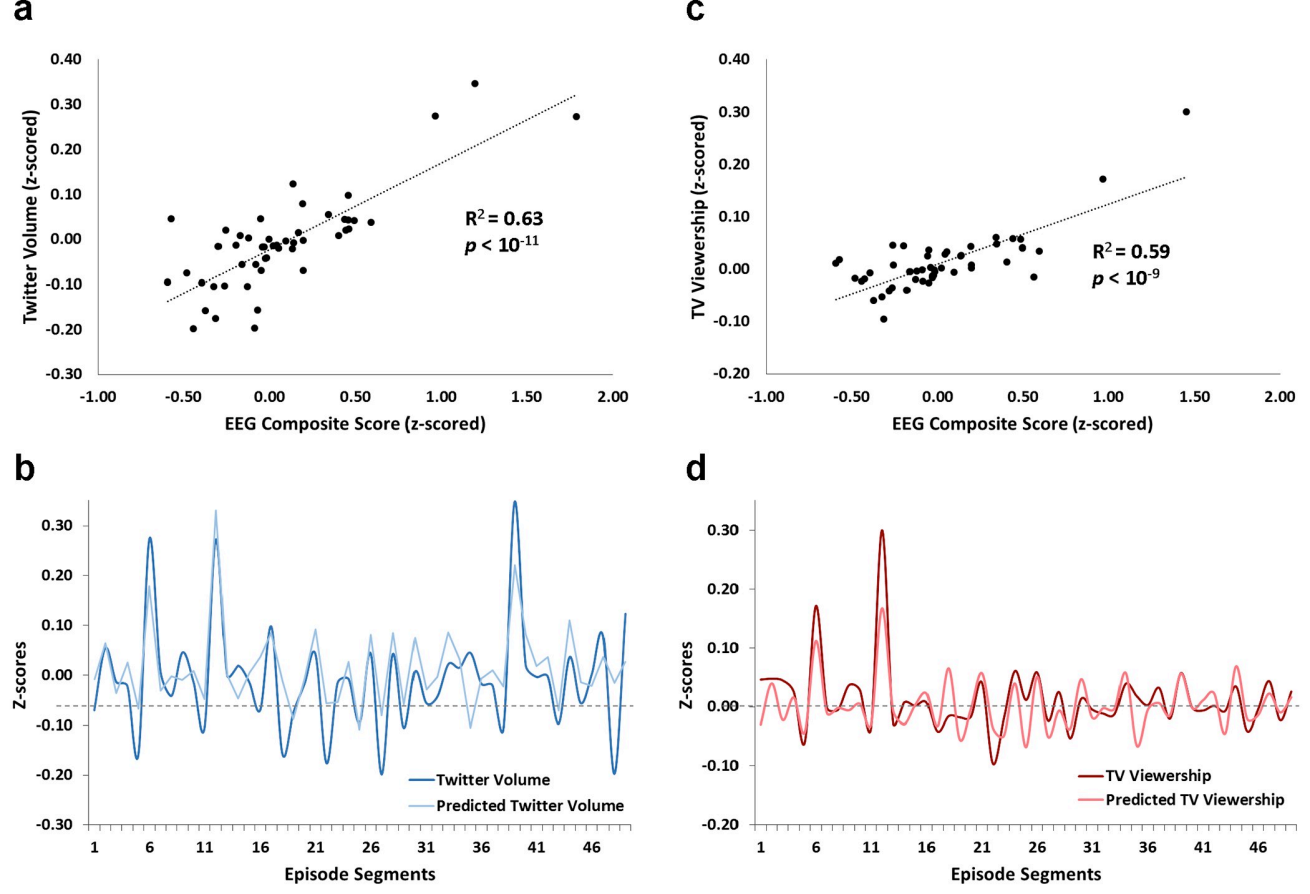
Composite EEG score predicts Twitter volume and TV viewership across program segments. (A) Correlation between minute-by-minute changes in the composite EEG score (consisting of a z-scored average of the fronto-central left-to-right alpha/beta asymmetry, alpha power decrease/theta increase, and theta and gamma power increases) and Twitter volume across discrete program segments (n = 49). (B) Minute-by-minute changes in Twitter volume are plotted against changes in Twitter volume predicted based on the composite EEG score. (C) Correlation between minute-by-minute changes in composite EEG score and TV viewership across discrete program segments (n = 49). (D) Minute-by-minute changes in TV viewership are plotted against changes in Viewership predicted based on the composite EEG score.

First, we examined whether individual frequency-based EEG metrics or the composite group-aggregate score can be used to predict changes in TV viewership and Twitter volume across program segments. The composite EEG score was calculated for each program segment by averaging segment values of the individual frequency metrics: the left-to-right fronto-central alpha/beta asymmetry (commonly associated with emotional motivation), decreases in the fronto-central alpha power and concomitant increases in theta power (commonly associated with attention processing), and increases in the fronto-central theta and gamma power (commonly associated with memory processing; see Methods). Two separate stepwise linear regression models were tested, in which the composite EEG score was used to estimate TV viewership or Twitter volume, respectively. Similarly, individual EEG scores were entered into two stepwise regressions predicting TV viewership or Twitter volume respectively (Table 2). A significant relationship was observed between the composite EEG score and Twitter volume; *R*^*2*^ = 0.63, *p* < 10^−11^ (Fig 2A and 2B). There was also a relationship between the composite EEG score and TV viewership; *R*^*2*^ = 0.57, *p* < 10^−9^ (Fig 2C and 2D). When examining contributions of individual EEG scores to Twitter volume and TV viewership, a similar pattern of results has emerged. In particular, all three independent variables were included in the respective stepwise regression models suggesting significant explanatory power of each individual EEG score. For Twitter volume, the order of variables entered into the final stepwise model was the alpha/theta power (attention), theta/gamma power (memory), and alpha/beta asymmetry (emotional motivation); *R*^*2*^ = 0.63 (adj. *R*^*2*^ = 0.61), *p* < 10^−5^. For TV viewership, the order of variables entered in the final stepwise model was the alpha/theta power (attention), alpha/beta asymmetry (emotional motivation), and theta/gamma power (memory); *R*^*2*^ = 0.68 (adj. *R*^*2*^ = 0.66), *p* < 10^−5^. These results indicate that EEG activity in a restricted sample of participants watching naturalistic TV stimuli can be successfully used to predict population-level behavior such as Twitter activity and TV viewership. Notably, predictive power of the single composite EEG score was comparable to the predictive power of all three individual EEG scores in combination, confirming the utility of both composite and individual EEG measures and suggesting that Twitter and viewership behaviors can be explained through the overall engagement of the audience.

**Table 2:** Regression results predicting TV viewership and Twitter volume.

Dependent Variable	Model	Independent Variable(s)	Model R^2^	Model Adj. R^2^	Model *p*-value	Beta Coefficient (standardized)	Beta *p*-value
**TV viewership**	LR	Composite EEG score	0.57	.57	<10^−5^	0.76	<10^−5^
**TV viewership**	SWMR	Full model	0.68	.66	<10^−5^		
		Alpha/theta power				1.07	<10^−5^
		Alpha/beta asym.				0.41	<10^−3^
		Theta/gamma power				0.30	0.003
**High TV viewership**^**a**^	SWMR	Full model	0.72	0.69	<10^−5^		
		Alpha/theta power				1.10	<10^−5^
		Alpha/beta asym.				0.52	0.002
**Low TV viewership**^**a**^	SWMR	Alpha/theta power	0.17	0.13	0.048	0.41	0.048
**Twitter volume**	LR	Composite EEG Score	0.63	0.62	<10^−5^	0.80	<10^−5^
**Twitter volume**	SWMR	Full model	0.63	0.61	<10^−5^		
		Alpha/theta power				0.71	<10^−5^
		Theta/gamma power				0.50	<10^−5^
		Alpha/beta asym.				0.39	<10^−3^
**High Twitter volume**^**a**^	SWMR	Alpha/beta asym.	0.48	0.44	<10^−3^	0.68	<10^−5^
**Low Twitter volume**^**a**^	SWMR	-	-	-	-	-	-
**TV viewership**	LR	Twitter volume	0.51	0.50	<10^−3^	0.72	<10^−5^
**TV viewership**	SWMR	Full model	0.67	0.66	<10^−5^		
		Composite EEG score				0.51	<10^−5^
		Twitter volume				0.40	0.001
**Twitter volume**	SWMR	Full model	0.67	0.66	<10^−5^		
		Composite EEG score				0.58	<10^−3^
		TV viewership				0.29	0.027

Adj.–adjusted, Asym.–assymmetry, LR–linear regression, SWMR–step-wise multiple regression.

^a^ For these analyses, TV viewership and Twitter volume values were split along the median for each variable and full step-wise multiple regression models were conducted on each median-split subgroup of data.

**Fig 3:**
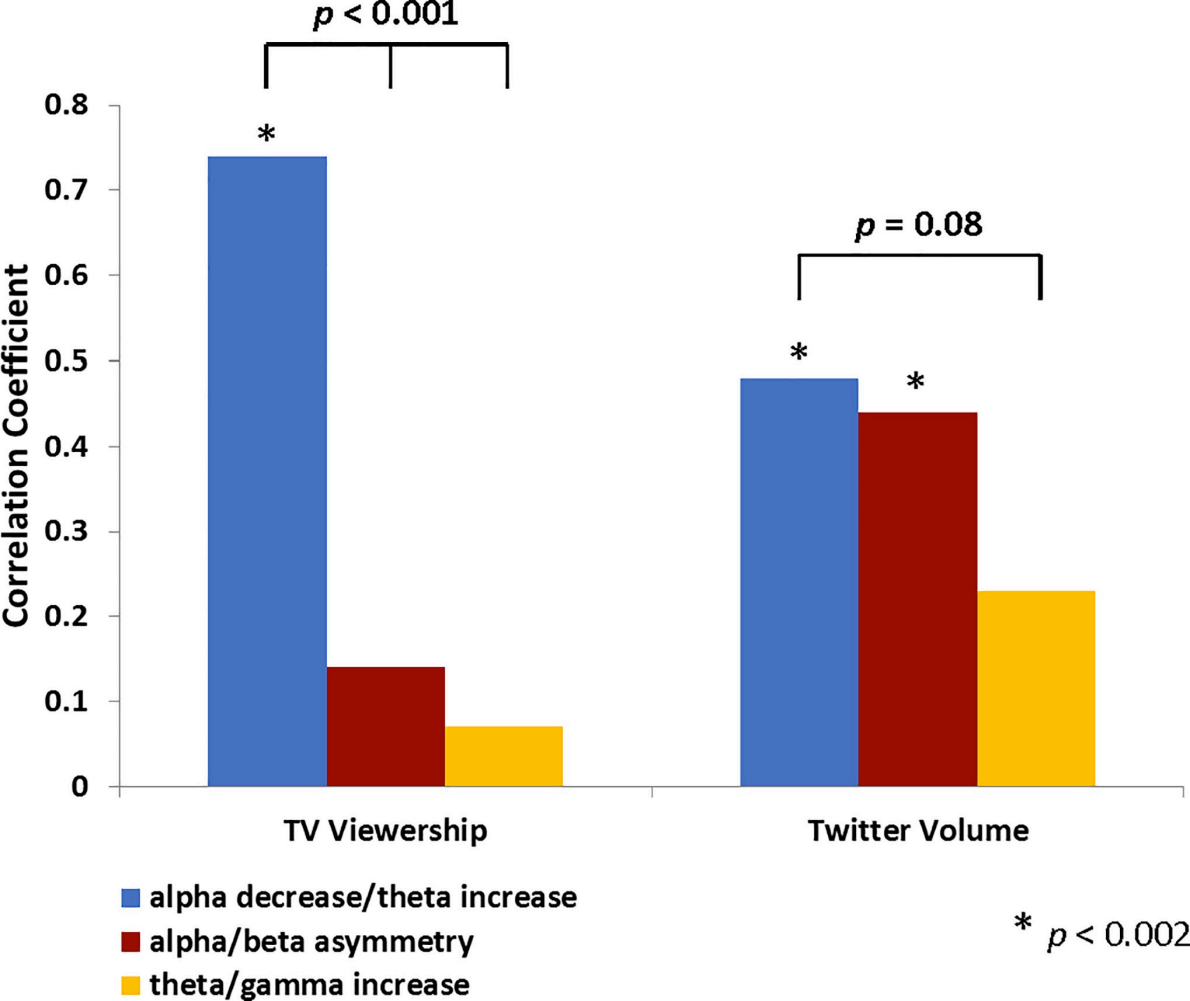
The relationship between individual EEG metrics and TV viewership, Twitter volume. Correlation coefficients were calculated to assess the relationship between individual EEG metrics (n = 49) and TV viewership as well as Twitter volume. Correlation coefficients were computed for fronto-central alpha power decreases/theta power increases typically associated with content-specific attention, alpha/beta asymmetry typically associated with motivational approach, and theta/gamma power increases typically associated with memory processing. The fronto-central alpha power decreases/theta power increases were significant predictors of minute-by-minute changes in both TV viewership and Twitter volume, while the alpha/beta asymmetry was exclusively linked with minute-by-minute changes in Twitter volume.

When the respective contribution of individual EEG scores to Twitter activity and TV viewership were examined, we observed that while all three EEG scores were entered into the respective stepwise regression models, not all variables had equal predictive power in isolation versus in combination with other variables. In particular, zero-order Pearson correlations between TV viewership and individual EEG scores revealed that fronto-central alpha power decrease/theta power increase (typically associated with attention allocation) was the best predictor of TV viewership (*r* = 0.74, *R*^*2*^ = 0.55, *p* < 10^−5^; Fig 3) relative to other frequency combinations, which did not have significant correlations with TV viewership and had significantly smaller correlation coefficients (alpha/beta asymmetry: *r* = 0.14, *p* = 0.34, *Z*_*difference*_ = 3.88, *p* < 10^−4^; theta/gamma increases: *r* = 0.07, *p* = 0.63, *Z*_*difference*_ = 4.22, p < 10^−5^, quantified by the Fisher’s z-to-r transform test for two correlation coefficients). For Twitter activity, alpha/theta power (associated with attention) had a significant zero-order correlation (*r* = 0.48, *R*^*2*^ = 0.23, *p* < 10^−3^) that was similar to the correlation between Twitter volume and the alpha/beta asymmetry score (associated with emotion motivation; *r* = 0.44, *R*^*2*^ = 0.19, *p* < 0.002; Fig 3). In fact, the fronto-central asymmetry score had higher correlation with Twitter Volume than TV viewership (*Z*_*difference*_ = 1.59, *p* = 0.05). The theta/gamma power score (associated with memory) did not have a strong correlation with Twitter activity (*r* = 0.23, *R*^*2*^ = 0.05, *p* = 0.11), with the trend difference in the magnitude of this correlation and correlations for alpha/beta asymmetry and alpha/theta power (*Z*_*difference*_ = 1.39, *p* = 0.08).

Given that the fronto-central alpha/beta asymmetry and theta/gamma power measures had significant contributions to the regression models but did have significant zero-order correlations with TV viewership and marginal correlations with Twitter activity (for theta/gamma power), these EEG scores are likely to reflect suppressor variables, indicating a potential interaction effect [115]. Detailed model analysis revealed that when TV episode segments were divided along the median split on TV viewership, the only EEG score with predictive power for segments with low TV viewership was the alpha decrease/theta increase; *R*^*2*^ = 0.17 (adj. *R*^*2*^ = 0.13), *p* = 0.048 (Table 2). In contrast, the stepwise regression model for segments with high TV viewership accounted for more variance and relied on both alpha/theta power as well as left-right alpha/beta asymmetry, in that order; *R*^*2*^ = 0.72 (adj. *R*^*2*^ = 0.69), *p* < 10^−5^. Similar analyses for Twitter activity revealed that no significant predictive model could be estimated for segments with low Twitter volume, while left-to-right alpha/beta asymmetry was the only EEG score that significantly predicted Twitter activity for segments with high Twitter volume; *R*^*2*^ = 0.47 (adj. *R*^*2*^ = 0.44), *p* < 10^−5^. These results indicate that while the alpha/theta power EEG score, commonly associated with attention processes, is required to predict TV viewership, higher levels of Twitter and TV utilization uniquely rely on the left-to-right alpha/beta asymmetry EEG score, commonly associated with emotion motivation.

Finally, we also observed a strong relationship between the two dependent outcome variables reflecting population-level behaviors—TV viewership and Twitter volume (*r* = 0.72, *R*^*2*^ = 0.52, *p* < 10^−5^; Table 2), which was expected, as real-time tweeting is only possible if people are watching the program. Given the significant regression models outlined above predicting TV viewership and Twitter volume based on EEG scores, we next examined whether the relationship between TV viewership and Twitter volume can be explained by changes in EEG activity elicited by program content. The partial correlation between Twitter volume and TV viewership, controlling for variance accounted by the composite EEG score, revealed a less robust link between the two dependent variables: *r* = 0.34, *R*^*2*^ = 0.12, *p* = 0.02. This decrease in the magnitude of the relationship between TV viewership and Twitter volume when controlled for EEG activity was statistically significant, *Z*_*difference*_ = 2.56, *p* = 0.01 (quantified by the Fisher’s z-to-r transform test for two correlation coefficients). This suggests a moderating effect, with significant proportion of shared variance in both TV viewership and Twitter volume explained by the overall neural engagement indexed by the composite EEG score.

To further examine whether EEG measures add any explanatory power above and beyond the reciprocal relationship between TV viewership and Twitter activity, we conducted separate step-wise regression analyses where TV viewership and Twitter activity were treated as either dependent or independent variable along with the composite EEG score (Table 2). For TV viewership, both Twitter volume and the composite EEG score were included in the final model, with the composite EEG score explaining more variance in TV viewership than Twitter volume; *R*^*2*^ = 0.67 (adj. *R*^*2*^ = 0.66), *p* < 10^−5^. Similarly, for Twitter volume, the composite EEG score and TV viewership were both included in the model, with the composite EEG score explaining more variance in Twitter activity than TV viewership; *R*^*2*^ = 0.67 (adj. *R*^*2*^ = 0.66), *p* < 10^−5^. These results indicate that EEG metrics add significant explanatory power to predictive models and that the composite EEG score is a better predictor of population-level behavior than either of the behavioral variables taken in isolation.

## Discussion

In this study, we examined whether brain activity, indexed by power changes in the theta, alpha, beta, and gamma EEG frequency bands, recorded in laboratory settings can be used to explain population-level success of TV shows, as measured by TV viewership and the extent of social media engagement with programming content. Three EEG score combinations were analyzed: decreases in the fronto-central alpha range concomitant with increases in theta power, commonly associated with attention processing [33, 63–64]; increases in the fronto-central theta and gamma power, commonly associated with memory processing [36, 76–77]; and the left-to-right fronto-central asymmetry in the alpha and beta frequency bands, commonly associated with emotional approach motivation [33, 53]. In addition, a single composite EEG score was derived by averaging the three individual EEG scores. We report several key findings that underscore the utility of EEG-derived metrics in predicting population-level behavioral engagement with TV programming.

Both the composite EEG score and the combination of all three individual EEG scores entered into a stepwise regression separately had significant correlations and accounted for over 60% of variance in TV viewership and Twitter activity across segments of the tested TV shows. Given that the three EEG measures have been extensively linked with attention, memory, and emotional motivation processing, these results suggest that TV programming performance is dependent on the overall cognitive and emotional engagement of audiences with the show content, as measured by EEG. These results are in line with previous behavioral and neurophysiological reports of the importance of these cognitive factors in perception and engagement with TV content [33, 76–77]. Notably, while there was a strong correlation between the two behavioral measures (i.e., Twitter activity and TV viewership) across episode segments, the composite EEG score was a better predictor of each of the population-level outcome variables than the other behavioral measure. Mediation analysis revealed that the composite EEG score explained a significant portion of the relationship between the two behavioral measures. The relationship between TV viewership and Twitter activity appears to be mechanistic–if more people are watching the show, it is likely that more people will be tweeting about it; and if more people tweet about it, then more audiences are likely to join [8, 14–16]. However, here we demonstrate that although there is a direct reciprocal connection between the two behaviors (e.g., audiences typically tweet about a show only if they are watching it), a large proportion of this relationship is accounted for by brain activity, reflecting attentional, memory, and motivational cognitive processes, that is elicited by TV programming. Specifically, we suggest that creative content that is more evocative, leading to higher levels of attentional focus, memory processing, and emotional motivation across audiences, will be more likely to keep viewers on the screen and may compel them to share their experiences with friends and family in real life or on social media. A key strength of the current study is the ability to link population-wise dynamics in TV content consumption based on brain activity of a select sample of participants. TV viewership and Twitter engagement depend on a wide variety of factors, ranging from current entertainment zeitgeist, to differences in airtime schedules, to population demographics (as seen in the top-level differences across episodes). However, show content is the core determinant of whether people tune in to watch a program or are compelled to tweet about it. Here we demonstrate that EEG-measured content-related brain activity explains up to two-thirds of overall variability in TV viewership and Twitter engagement.

Although all three EEG metrics had significant contributions to the predictive models, not all of them had the same pattern of relationship with the two outcome variables. Among the three EEG metrics, TV viewership (overall or median-split) was best correlated with the fronto-central decreases in alpha and increase in theta power, indexing attentional processing, suggesting that TV content must attract and sustain viewers’ attention in order to retain the audience. While the left-to-right asymmetry, commonly associated with emotional approach motivation and positive affect, was not a significant predictor of TV viewership overall or for segments with low viewership, it was the second highest predictor for show segments with high audience viewership, indicating that approach motivation is an important factor in audiences staying with the show once they are already engaged the content. Emotional motivation also played a key role in predicting Twitter activity. Specifically, the left-to-right asymmetry was the second highest predictor for Twitter volume overall and the only significant predictor for segments with high median-split Twitter activity. These findings indicate that TV shows not only have to attract audience attention but also evoke strong emotional responses in order to compel viewers to share their thoughts and impressions on social media. Importantly, it is not enough for the show content to simply portray emotions or feelings. Previous research indicates that fronto-central alpha asymmetry is only detected when viewers internalize and subjectively express and feel the emotion themselves [59]. Thus, it is the subjective experience of emotion, as measured by the alpha/beta EEG asymmetry, that is likely to be a mediating factor in the relationship between programming content and show-specific social media engagement. Notably, the EEG measure of emotional motivation was particularly important for segments with high viewership or Twitter volume, indicating a break-through threshold above which emotional and cognitive engagement with the show is more closely linked with audience behavior than show segments with low audience participation. Specifically, the variability across segments with low TV viewership or Twitter volume is more likely to be dominated by noise and may be more readily explained by whether audiences are simply paying attention or not. In contrast, viewership and Twitter activity above a particular threshold are more likely to have higher signal-to-noise ratio and be influenced by subjective experiences of the creative content rather than extraneous noise factors. Thus, while EEG measures of attention can serve as a minimum necessary (e.g., gating) metric for predicting TV consumption behaviors, the emotional motivation measures appear to reflect a break-through potential for TV programming–attention is necessary for audiences to be aware of the content, but emotional motivation is what is going to prompt pro-active engagement with the content. These findings are also consistent with previous reports indicating that the desire to express feelings and emotional responses in response to what is happening on the screen is a key factor in why people tweet [10, 12]. For example, Wohn and Na found that over 60% of program-specific tweets were expressing subjective opinions, with about a quarter of tweets explicitly containing emotional language [12]. Although these percentage values could vary from show to show, they indicate the extent to which emotional engagement is important for tweeting behavior. Future research may use EEG measures to examine a potential link between viewers’ experienced emotional responses, the content of tweets, and the propensity to stay with the show.

In the current study, fronto-central hemispheric lateralization was more closely linked with proactive behavior (i.e., tweeting), which is in line with the approach motivation interpretation of this EEG metric [58–62]. However, it would be erroneous to posit that emotional motivation is less important in attracting viewers to watch the TV content. The current study was designed as a cross-sectional analysis of continuous watching of a single TV episode. Thus, we were not able to assess whether EEG-indexed changes in emotional motivation, attention, or memory may correlate with individuals’ desire to watch the next episode of the show. A longitudinal design, where multiple episodes across a single show are examined (serial episodes shown weekly or continuously in online-streaming of consecutive episodes), could determine whether motivational approach predicts TV viewership when proactive behavior is measured (i.e., increase or decrease in viewership from episode to episode of a single show). Similarly, memory processes are likely to play a more prominent role in sustaining viewership across multiple episodes, especially when they are spread out over time vs. watched continuously.

The current findings are in line with previous reports of overall EEG synchrony across subjects correlating with fluctuations in TV viewership and the number of tweets for a single TV show episode or across TV commercials [99]. Similar single-score EEG measures have been found to be effective in predicting movie performances based on tested movie trailers [29–31]. Single-score EEG measures have also been correlated with memory for creative content of TV advertisement [36] and in-market performance of tested TV ads [45]. Interestingly, all these composite or single-score EEG measures have been based on different features of the EEG signal. The neural similarity score derived by Barnett and Cerf was calculated in the alpha frequency band [29]. Boksem and Smidts as well as Christoforou and colleagues found that the best predictor of movie performance in wide release was the power in the beta and gamma frequency ranges over the frontal sites [30–31]. Dmochowski and colleagues found that inter-subject temporal reliability of the EEG signal in the low frequencies, especially within the 8–10 Hz alpha frequency range over the fronto-central sites, was predictive of preference, viewership and Twitter activity for the *Walking Dead* pilot as well as Super Bowl ads [99]. Kong and colleagues correlated TV commercial memorability with a composite EEG metric (the impression index) based on the power within theta and alpha frequency bands [36]. Finally, Venkatraman and colleagues examined in-market prediction performance of a composite EEG metric consisting of the left-to-right frontal asymmetry and power over the occipital sites in the alpha frequency band [45]. Some of these EEG measures were derived empirically by searching for the most predictive combination across frequency bands or scalp locations [30–31, 99], while other studies, including the one presented herein, relied on hypothesis-driven derivation of EEG measures that was based on previous literature [29, 36, 45].

Despite the mentioned differences in composition and derivation of these EEG metrics, all of them have been found to correlate with behavioral measures of efficacy of the creative content, which is not surprising given that many of these measures rely on common features of the EEG signal. For example, a decrease in alpha power, indicating attentional processing, has been used as a base or a component of single-score EEG measures across many of studies [29, 36, 45, 99]. As we demonstrate here, attention processing, instantiated in the power fluctuations within the alpha and theta frequency bands, is the most common predictor of both TV viewership and Twitter activity and is often the minimum necessary cognitive process associated with successful stimulus perception and behavioral output [116]. And since most consumer behaviors rely on more than one cognitive process, it is expected that EEG measures that use signal features associated with other cognitive processes will also correlate with behavioral outcomes in these studies. In fact, some of the variability in the strength of the relationship between EEG metrics and outcome variables, as well as which features of the EEG signal are found to be most predictive, can be explained by the choice of the behavioral outcomes. It is reasonable to hypothesize that different performance criteria (e.g., box office ticket sales, Twitter volume, TV viewership, purchase intent, etc) will rely on different cognitive processes to various degrees and, thus, will be reflected in different features of the EEG signal. Similarly, while we found in the current study that the single-score EEG metric was similar in its predictive power to the three individual EEG measures that were also represented in the single-score metric, we would not expect the same concordance between the single-score measure presented herein and individual EEG measures that index other cognitive processes, such as surprise or implicit associations. Finally, some differences in results among existing studies could also be explained by the choice of EEG features used to index specific cognitive processes. For example, many studies use alpha desynchronization alone as a measure of attentional focus [29, 36, 45]. However, attentional processing has been associated with changes not only in the alpha power but also in the power within the theta frequency band [116–117]. This could account for the increased explanatory power of the attention metric in the current study, which is similar in its composition to the impression index proposed by Kong and colleagues [36]. Thus, prediction accuracy for both single-score and individual EEG measures undoubtedly depends on the careful selection of component features of the EEG signal.

Although single-score EEG metrics are simple to use and often correlate with and are able to accurately predict consumer behavior, they are also limited in their explanatory depth and scope. The validity and usability of single-score EEG metrics are contingent on their component elements–the more features that reflect diverse cognitive processes are used, the more accurate such composite metrics will become. However, we also demonstrate that the use of individual EEG metrics reflecting specific cognitive and emotional processes provides a more rich and nuanced examination of different aspects of consumer behavior.

Interpretation of the current study results should be taken cautiously in light of several limitations. First, as it is common in consumer neuroscience research, we employed the reverse inference approach. Specifically, we did not directly manipulate stimulus properties to interrogate a specific cognitive process. Rather, we relied on extensive literature repeatedly detailing associations between changes in EEG power in particular frequency bands and corresponding cognitive processes, including attention, memory, and motivation. As discussed above, the exact composition of EEG metrics that are taken to index each of these cognitive processes is likely to affect accuracy and reliability of obtained results. It is also possible that other cognitive processes may have contributed to changes in EEG power within specific frequency bands; for example, anxiety and cognitive control are sometimes associated with increased midline theta [118]. However, given the extant literature, the effect of these secondary contributions to current results is likely to be minor. Second, the design of the current study was cross-sectional, where a single episode of each TV show was examined. This approach has considerable benefits since it allows generalizability across shows of various genres, popularity, broadcast characteristics, etc. However, we did not test whether EEG measures in response to a single episode (such as a pilot) can be used to predict TV show viability (viewership and social media engagement) for subsequent episodes. Several previous studies have demonstrated that such predictions are possible by linking EEG activity in response to movie trailers with subsequent box office performance of the corresponding movies [29–31]. Our study serves as a proof of concept that EEG measures can be successfully used to assess performance of TV programming, and future research is needed to establish longitudinal prediction value of EEG indices of attention, memory, emotional motivation, and overall engagement. It is also unknown whether the success of all television show genres relies on similar cognitive and emotional processes or whether we are likely to find different patterns of EEG responses, indicating different weights for attention, memory, or motivation, for documentaries vs. dramas vs. reality TV. Finally, the current study used population-level behaviors as the output variables. We did not assess whether EEG measures correlated with viewers’ subjective self-reported opinions about and preferences towards the tested show episodes. Future research should address the above questions and aim to replicate and extend these findings, providing more in-depth analysis of core factors that contribute to TV show success.

In summary, we demonstrate that the best predictors of TV viewership and Twitter engagement for prime-time TV shows are a combination of attention, emotional motivation, and memory processes. Attentional focus towards the show content translated to both continuous single-episode TV viewership and greater Twitter activity. In contrast, emotional motivation processes were critical for Twitter activity but not TV viewership. These results reveal that successful TV shows are able to capture viewers’ attention and evoke positive emotional responses and demonstrate that EEG-derived measures of specific cognitive processes can predict success or failure of TV content and can be used to predict naturalistic population behaviors.

## Supporting information

S1 Dataset Minute-by-minute EEG, Twitter volume, and TV viewership values for each TV show episode tested. Each spreadsheet in the Excel file corresponds to one of the tested episodes and contains minute-by-minute values Twitter volume (raw), TV viewership (raw), and EEG metrics (pre-processed) associated with Attention, Motivation, and Memory, as well as the composite EEG metric (the average of the other three metrics). Ad breaks and missing values are indicated in the Notes field on each spreadsheet.
(XLSX)
